# Predictable Changes in Eelgrass Microbiomes with Increasing Wasting Disease Prevalence across 23° Latitude in the Northeastern Pacific

**DOI:** 10.1128/msystems.00224-22

**Published:** 2022-07-20

**Authors:** Deanna S. Beatty, Lillian R. Aoki, Brendan Rappazzo, Chelsea Bergman, Lia K. Domke, J. Emmett Duffy, Katie Dubois, Ginny L. Eckert, Carla Gomes, Olivia J. Graham, Leah Harper, C. Drew Harvell, Timothy L. Hawthorne, Margot Hessing-Lewis, Kevin Hovel, Zachary L. Monteith, Ryan S. Mueller, Angeleen M. Olson, Carolyn Prentice, Fiona Tomas, Bo Yang, John J. Stachowicz

**Affiliations:** a Department of Evolution and Ecology, University of California, Davis, California, USA; b Data Science Initiative, University of Oregon, Eugene, Oregon, USA; c Department of Ecology and Evolutionary Biology, Cornell University, Ithaca, New York, USA; d Department of Computer Science, Cornell University, Ithaca, New York, USA; e Department of Biology and Coastal & Marine Institute, San Diego State University, San Diego, California, USA; f College of Fisheries and Ocean Sciences, University of Alaska Fairbanks, Juneau, Alaska, USA; g MarineGEO Program and Smithsonian Environmental Research Center, Edgewater, Maryland, USA; h Biology Department, Bowdoin College, Brunswick, Maine, USA; i Department of Sociology and College of Sciences GIS Cluster, University of Central Florida, Orlando, Florida, USA; j Nearshore Marine Ecology, Hakai Institute, Heriot Bay, British Columbia, Canada; k Institute for the Oceans and Fisheries, University of British Columbia, Vancouver, British Columbia, Canada; l Department of Microbiology, Oregon State University, Corvallis, Oregon, USA; m Instituto Mediterráneo de Estudios Avanzados (UIB-CSIC), Esporles, Spain; n Department of Urban and Regional Planning, San Jose State University, San Jose, California, USA; University of Technology Sydney

**Keywords:** seagrass, *Zostera marina*, *Labyrinthula zosterae*, phyllosphere, microbiome, wasting disease

## Abstract

Predicting outcomes of marine disease outbreaks presents a challenge in the face of both global and local stressors. Host-associated microbiomes may play important roles in disease dynamics but remain understudied in marine ecosystems. Host–pathogen–microbiome interactions can vary across host ranges, gradients of disease, and temperature; studying these relationships may aid our ability to forecast disease dynamics. Eelgrass, *Zostera marina*, is impacted by outbreaks of wasting disease caused by the opportunistic pathogen *Labyrinthula zosterae*. We investigated how *Z. marina* phyllosphere microbial communities vary with rising wasting disease lesion prevalence and severity relative to plant and meadow characteristics like shoot density, longest leaf length, and temperature across 23° latitude in the Northeastern Pacific. We detected effects of geography (11%) and smaller, but distinct, effects of temperature (30-day max sea surface temperature, 4%) and disease (lesion prevalence, 3%) on microbiome composition. Declines in alpha diversity on asymptomatic tissue occurred with rising wasting disease prevalence within meadows. However, no change in microbiome variability (dispersion) was detected between asymptomatic and symptomatic tissues. Further, we identified members of Cellvibrionaceae, Colwelliaceae, and Granulosicoccaceae on asymptomatic tissue that are predictive of wasting disease prevalence across the geographic range (3,100 kilometers). Functional roles of Colwelliaceae and Granulosicoccaceae are not known. Cellvibrionaceae, degraders of plant cellulose, were also enriched in lesions and adjacent green tissue relative to nonlesioned leaves. Cellvibrionaceae may play important roles in disease progression by degrading host tissues or overwhelming plant immune responses. Thus, inclusion of microbiomes in wasting disease studies may improve our ability to understand variable rates of infection, disease progression, and plant survival.

**IMPORTANCE** The roles of marine microbiomes in disease remain poorly understood due, in part, to the challenging nature of sampling at appropriate spatiotemporal scales and across natural gradients of disease throughout host ranges. This is especially true for marine vascular plants like eelgrass (*Zostera marina*) that are vital for ecosystem function and biodiversity but are susceptible to rapid decline and die-off from pathogens like eukaryotic slime-mold *Labyrinthula zosterae* (wasting disease). We link bacterial members of phyllosphere tissues to the prevalence of wasting disease across the broadest geographic range to date for a marine plant microbiome-disease study (3,100 km). We identify Cellvibrionaceae, plant cell wall degraders, enriched (up to 61% relative abundance) within lesion tissue, which suggests this group may be playing important roles in disease progression. These findings suggest inclusion of microbiomes in marine disease studies will improve our ability to predict ecological outcomes of infection across variable landscapes spanning thousands of kilometers.

## INTRODUCTION

Host-associated microbiomes are vital to maintaining marine ecosystem function and biodiversity (1), but relatively little is known about how marine disease outbreaks affect these relationships (2–4). Host-associated microbes play important roles in nutrient cycling by providing limiting nutrients and substrates to their hosts (5–7). They can also reduce colonization or proliferation of pathogens through resource competition and by production of allelochemicals (5–8). Beneficial microbes may improve host resilience to abiotic or biotic stressors (6). While host microbiomes can acclimate to variable environments, whether these changes are adaptive is often unclear (9, 10). Local and global stressors such as pollution, overfishing, and temperature anomalies can disrupt marine microbiomes (5). For example, overfishing and thermal stress can interact to destabilize coral microbial communities, and this increasing microbial beta diversity is associated with higher rates of coral disease and mortality (11). Further, stressors, like warming ocean temperatures, may make hosts more susceptible to pathogen attack or increase the geographic range, abundance, or virulence of pathogens (12, 13). Primary pathogens are known for some marine diseases; however, in many cases pathogens remain unidentified (2, 4, 14). Rather than a single pathogen, microbial consortia may also be responsible for disease states. In these cases, a broader screening approach may be needed to identify the microbes involved in disease progression (3, 4). Further, we have a limited understanding of how marine microbiomes vary, particularly along extensive environmental gradients throughout large host ranges and with disease states (4, 6, 15, 16).

Eelgrass (*Zostera marina*) is a widespread seagrass species throughout the northern hemisphere and is susceptible to attack by the opportunistic pathogen *Labyrinthula zosterae*, a colonial protist that causes eelgrass wasting disease (17, 18). *L. zosterae* degrades plant tissues, which leads to black-edged necrotic lesions and can result in plant mortality in severe cases (17, 19). The first documented outbreak of eelgrass wasting disease was in the North Atlantic during the 1930s and led to catastrophic losses of eelgrass and declines of associated fisheries (14, 19–21). Recurring disease outbreaks are linked to eelgrass decline (22, 23). Light limitation and warmer temperatures are implicated in host susceptibility to this ubiquitous marine pathogen, but low salinity may mitigate these effects (18, 24). Further, prevalence of infections can reach 79–96% during summer months (25, 26). Recently, Groner et al. (23) linked warmer temperatures during the eelgrass growing season to increased prevalence of wasting disease in the San Juan Islands, Washington. Aoki et al. (27) observed similar links between warm thermal anomalies and increased wasting disease prevalence across 23° latitude in the Northeast Pacific. Although experimental studies show that warmer temperatures enhance *L. zosterae* growth and abundance (28), we do not yet know how eelgrass microbiomes vary along temperature gradients and with fluctuating disease prevalence in natural meadows.

*Zostera*-associated microorganisms could promote plant resilience to abiotic (e.g., temperature anomalies) and biotic stressors (e.g., pathogenic microbes) as observed in terrestrial plants (29, 30). For example, *Zostera* bacterial associates can benefit their hosts by fixing nitrogen, detoxifying rhizomes of hydrogen sulfide, and producing agarases that can break down fouling epiphytes (7, 31, 32). Cultured isolates of Z. *marina* leaves produce algicidal and algal growth inhibiting compounds that may prevent fouling and help regulate the phyllosphere microbiome (33). Alternatively, members of the microbiome may facilitate pathogens; for example, manipulation of eelgrass leaf microbiomes with antibiotics or dilute bleach prior to inoculation with *L. zosterae* can lead to less severe wasting disease lesions (Graham et al., submitted for publication). Thus, there are large knowledge gaps in how eelgrass-associated microbes interact to facilitate or inhibit pathogens and how these interactions may vary with environmental conditions.

**FIG 1:**
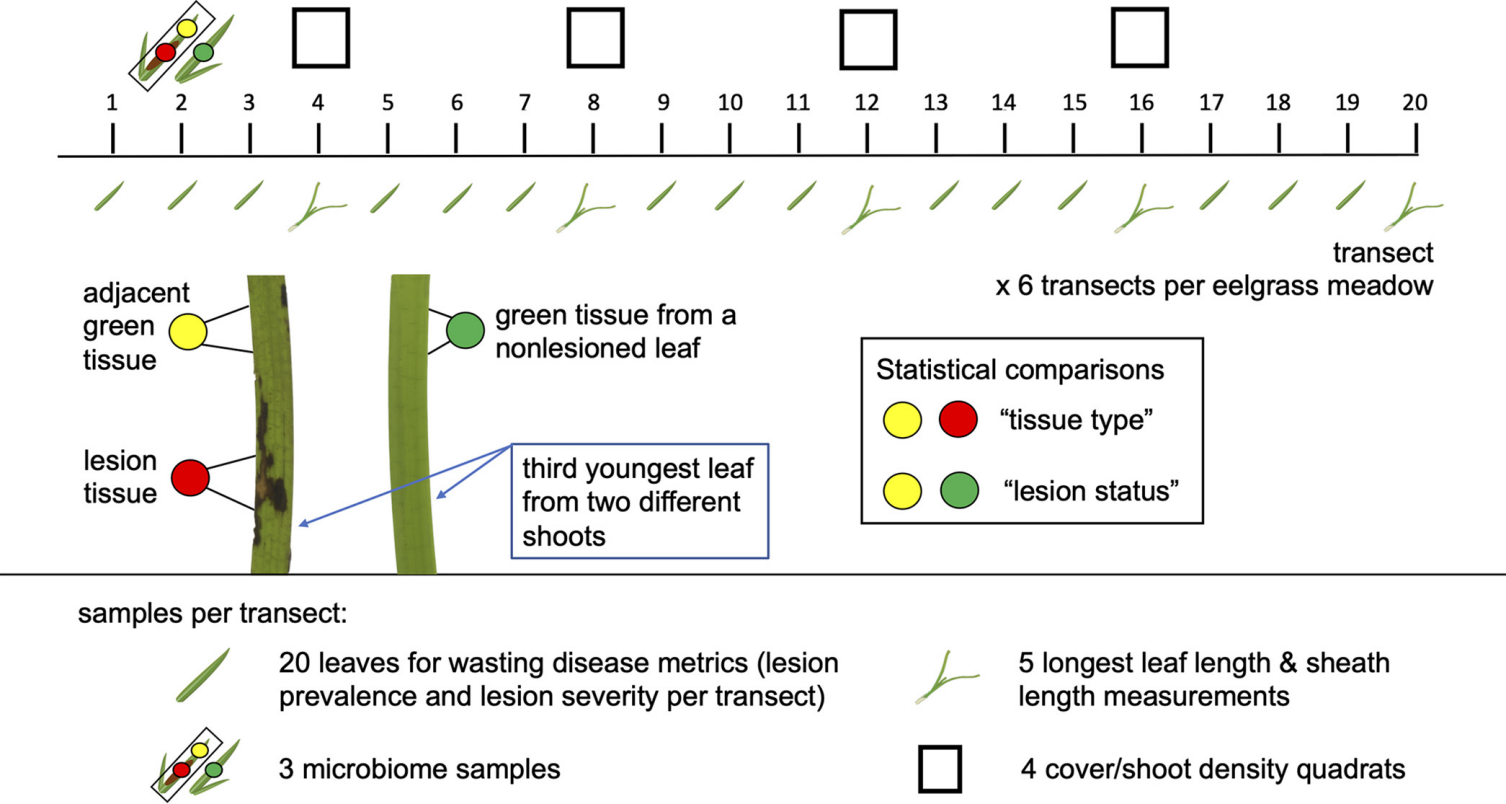
Depiction of the sampling design within each meadow. We sampled six transects per meadow to characterize the prevalence and severity of wasting disease lesions and meadow characteristics (longest leaf length, sheath length, and shoot density). We collected three microbiome samples per transect. This included lesion tissue and green tissue adjacent to lesions from the same leaf for comparisons of “tissue type.” We also sampled green tissue from a nonlesioned leaf from a different shoot for comparisons of “lesion status.” Lesion status allows comparisons among green tissue samples from both lesioned and nonlesioned leaves.

Predicting marine disease dynamics in a changing ocean will require investigation of host–pathogen–microbiome interactions across environmental gradients and varied levels of disease over large spatial scales (3, 4, 16). Effects of *L. zosterae* on eelgrass can differ across local to continental scales (22, 25, 26), and little is known about how host microbiomes change with disease prevalence or severity. Only one study to date investigated eelgrass microbiomes throughout the host's range but did not explore how microbial communities may vary with disease (34). Here we investigate how the *Z. marina* phyllosphere microbiome varies with wasting disease prevalence (via presence or absence of lesions on leaves) and severity (lesion area relative to total leaf area) within meadows across 23° latitude in the Pacific Northeast (see sampling design in Fig. 1). We test the following hypotheses: (i) phyllosphere microbial communities differ between wasting disease lesioned and nonlesioned leaves (lesion status, Fig. 1) and between lesion and adjacent green tissue (tissue type, Fig. 1) from lesioned leaves in consistent ways across regions; (ii) due to their ability to respond rapidly to environmental and biotic change, phyllosphere microbial taxa from asymptomatic tissue may exhibit changes in alpha or beta diversity as wasting disease prevalence or severity increases across meadows in our study. We also compare the effect of wasting disease with that of other potential factors, including temperature and eelgrass morphology, that may vary with phyllosphere and seawater microbial communities.

## MICROBIOME DATA ANALYSES

### Data preparation.

We trimmed sequence read ends with low quality scores (Phred score cutoff 25), removed Nextera adapters, and filtered out trimmed reads with fewer than 20 bp length using Trim Galore! software (http://www.bioinformatics.babraham.ac.uk/projects/trim_galore/). We merged trimmed read pairs with FLASH (35) with a minimum overlap of 20 bp. We imported merged reads into QIIME2 version 2020.2 (36) using a manifest file. We used Deblur (37) in QIIME2 to remove erroneous sequences, trim sequences to 390 bp (median quality score was 38 for the entire length of 390 bp), remove sequence variants with fewer than 10 occurrences across all samples, and remove chimeras. From 44,847,333 total sequences generated, 9,248,489 sequences remained (21% of total sequences) following Deblur filtration steps. We trained a taxonomic classifier with a Naïve Bayes model within QIIME2 on SILVA v.138.1 99% similarity 16S rRNA reference database trimmed with our primer pair (F515 and R926) and dereplicated within QIIME2 to improve computation time (38, 39). We classified exact sequence variants with sklearn within QIIME2 using the function qiime feature-classifier classify-sklearn (38, 39). Following classification of sequence variants, we removed mitochondria and chloroplasts with QIIME2 taxa filter-table script. Following this filtration step, 5,657,718 total sequences remained across all samples, with sequence counts ranging from 2 to 33,730 sequences per sample. Given the large variation in sequence counts across samples, we rarefied sequence variant tables to 4,660 sequences per sample prior to performing alpha and beta diversity analyses and random forest regression analyses to avoid confounding sequence count with experimental predictors of interest (40, 41). This rarefaction depth permitted retention of 40% of our reads and 75% of our samples. We imported sequence count tables, taxonomy, and metadata into R v1.2.5042 (42) with package qiime2R v0.99.34 (https://github.com/jbisanz/qiime2R) for use with R package phyloseq v1.3.4 (43) and vegan v2.5.6 (44).

### Microbial community beta diversity analysis.

We calculated Bray Curtis dissimilarity of microbial communities associated with eelgrass and seawater with the R package phyloseq (43), and performed principal coordinate ordination (PCoA) on resulting dissimilarity matrices to visualize differences between samples in multidimensional space and in response to our predictor variables. We performed multivariate analysis of variance (45, 46) with the “adonis2” function R package phyloseq (43) to test for effect sizes and significance of predictor variables on Bray Curtis dissimilarity matrices from phyllosphere microbial community tables of green tissue samples from both lesioned and nonlesioned leaves at the transect level. Predictor variables included lesion prevalence, lesion severity, lesion status, tidal height, leaf area, shoot density, sheath length, longest leaf length, sea surface temperature (SST) on the day of sampling, 30-day maximum and minimum SST prior to sampling, and locations of meadows nested within each region. We also tested for significance of these predictor variables with the “adonis2” function after randomly shuffling the order of predictor variables because precedence of factors can influence test results. Due to lack of independence between lesion tissue and adjacent green tissue sampled from the same leaf, we chose to subsequently run a separate beta diversity analysis on these samples to test for effects of tissue type on microbial community composition using the Bray Curtis dissimilarity metric and the adonis2 function described above. We used the function “betadisper” in R package vegan 2.5.6 (44) to test for differences in microbial dispersion, a value calculated by per sample distance from the centroid for any grouping variable in ordination space. Greater dispersion in ordination space means that microbial communities are more variable, or in other words exhibit greater microbial beta diversity, for one level of a factor compared to another level of a factor (47). We performed tests of microbial dispersion for the following factors: region of sample collection, lesion status for green tissue samples, and tissue type on lesioned leaves. After observing a large effect size of the region of sample collection on microbial community composition, we similarly tested for effects of disease metrics on microbiome composition and dispersion within each region separately for green tissue from both lesioned and nonlesioned leaves. Lastly, we tested for effect sizes and significance of predictor variables on Bray Curtis dissimilarity matrices from seawater microbial community tables at the site level. Predictor variables included lesion prevalence, lesion severity, leaf area, shoot density, sheath length, longest leaf length, SST on the day of sampling, 30-day maximum and minimum SST prior to sampling, and locations of meadows nested within each region.

### Microbial community alpha diversity analysis.

We used the phyloseq estimate_richness function to estimate alpha diversity metrics, sequence variant richness, and Shannon diversity (43). We used generalized additive mixed effect models within R package mgcv v1.8.33 (48, 49) to test for effects of predictor variables on alpha diversity values at the transect level for eelgrass microbiomes. Predictor variables included lesion prevalence, lesion severity, lesion status, tidal height, shoot density, sheath length, SST on the day of sampling, and region on negative binomial distributed sequence variant richness and gaussian distributed Shannon diversity values for eelgrass microbiomes from green tissue samples from lesioned and nonlesioned leaves. In each alpha diversity model (Shannon diversity and sequence variant richness), meadow location was a random effect, and a thin plate regression spline smoothing function was applied to lesion prevalence due to nonlinearity of this explanatory variable.

Due to lack of independence between lesion tissue and adjacent green tissue sampled from the same leaf, we chose to subsequently run a separate alpha diversity analysis on these samples from the analysis described above. We tested for effects of tissue type, i.e., lesion versus adjacent green tissue, on sequence variant richness and Shannon diversity by building generalized additive mixed effect models (48, 49). In each model, meadow location was a random effect, and we applied thin plate regression spline smoothing functions for sheath length in the richness model and sheath length and lesion prevalence in the Shannon diversity model. Factors included lesion prevalence, lesion severity, tissue type, tidal height, shoot density, sheath length, SST on the day of sampling, and region. Our Shannon diversity model included an interaction term between region and lesion severity following observation of a pattern in the data between these factors suggestive of a potential interaction of these factors on Shannon diversity.

We used linear mixed effects models to test for effects of predictor variables on sequence variant richness and Shannon diversity for seawater microbial communities at the site level within R package nlme v3.1.150 due to the linear relationship between the response and predictor variables (50). Factors included lesion prevalence, lesion severity, shoot density, sheath length, SST on the day of sampling, and region, with a random effect of meadow location. We modeled exponential variance structures for lesion severity and SST to account for variance patterns in the data.

### Random forest analysis.

To identify family-level taxa that are predictive of lesion prevalence per transect, we used a supervised learning regression, random forest (39, 51), in QIIME2 with 1,000 decision trees to train a predictive model following rarefaction to 4,660 sequences per sample. Sequence tables were collapsed at the taxonomic level family with QIIME2 function taxa collapse prior to running random forest regression. Twenty percent of green tissue samples from lesioned and nonlesioned leaves were randomly selected to train the model. We tested for correlations between taxa relative abundances with lesion prevalence per transect for the top 10 predictive taxa from green tissue samples using a custom-written function “correlate” that wraps cor.test from the R package stats v4.0.2 (42) in order to test multiple taxonomic groups. After failing to detect significant correlations for three of the top 10 predictive bacterial families, we tested for correlations between relative abundances of bacterial families from either lesioned or nonlesioned leaves separately with lesion prevalence per transect. We plotted relative abundances of predictive taxa from green tissue samples that were significantly correlated with lesion prevalence per transect with R package ggplot v3.3.5 (52).

We similarly ran random forest regression at the family level to identify microbial predictors of the maximum SST from a 30-day window prior to sampling. This allowed us to determine if temperature and wasting disease prevalence influenced the same microbial taxa, given that SST could directly affect microbes or could indirectly affect microbes mediated through wasting disease on leaves. We tested for correlations between the relative abundances of the top 10 predictive taxa with site level 30-day maximum SST using the custom written function “correlate” that wraps cor.test from R package stats v4.0.2 (42), as described above. We plotted predictive taxa significantly correlated with 30-day maximum SST by their relative abundances in R package ggplot v3.3.5 (52). After failing to detect significant correlations for two of 10 predictive bacterial families, we tested for correlations between relative abundances of bacterial families from either lesioned or nonlesioned leaves separately with 30-day maximum SSTs.

### Differential relative abundance testing.

We tested for differences in relative abundances of sequence variants and family-level taxa between green tissue from lesion and nonlesioned leaves (lesion status) and, separately, between lesion and green tissue adjacent to lesions (tissue type) with DESeq function in R package DESeq2 v1.30.1 (53). By testing at both the sequence variant and family level, we can better understand the distribution and relative abundances of enriched families and, potentially, sequence variants across our geographic range. Deseq2 performs an internal normalization procedure prior to differential abundance testing across grouping variables (54). However, samples with very low sequence counts (<1,000 sequences per sample) can still confound results for normalization procedures other than rarefaction (40). Thus, we imported unrarefied sequence count tables into DESeq2 after removing samples with low sequence counts (<1,000 sequences per sample). We performed differential abundance analysis on negative binomial distributed data using Wald significance tests, parametric fit of dispersions, and poscounts for estimation of size factors, which is appropriate for sequence count data.

## RESULTS

### Beta diversity results.

**FIG 2:**
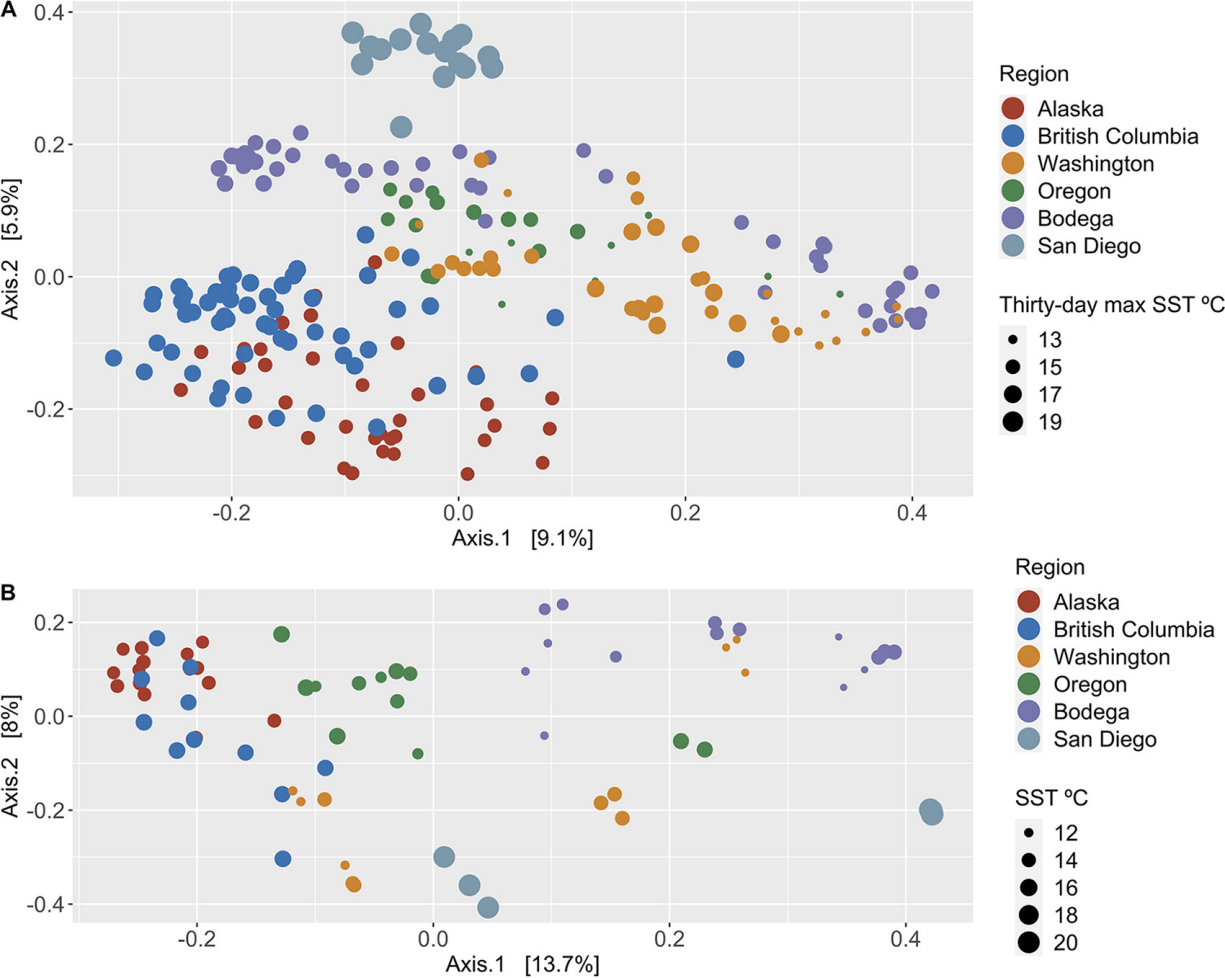
Microbial community composition differs predominantly by geographic region for both (A) eelgrass green tissue (from lesioned and nonlesioned leaves) and (B) seawater. Following regional effects, the factors that best explain eelgrass microbial community composition are 30-day maximum sea surface temperature in Celsius (4%), and prevalence of wasting disease lesions within meadows (3%). The factors that best explain seawater microbial community composition following regional effects are severity of wasting disease lesions (5%), eelgrass leaf area (5%), longest leaf length (5%), and sea surface temperature metrics in Celsius (4%). *P* values and effect sizes for all factors can be found in Table 1A and B. Sequence variant tables were rarefied to 4,660 sequences per sample prior to calculating Bray Curtis dissimilarity and performing principal coordinate analysis.

Eelgrass and seawater microbial communities differed strongly among geographic regions (Fig. 2A and B, PERMANOVA, permutation multivariate analysis of variance; Table 1A and B; Fig. S1 at https://doi.org/10.6084/m9.figshare.20097290.v1; Fig. S2 at https://doi.org/10.6084/m9.figshare.20097302). After accounting for these regional differences, disease metrics and meadow characteristics still significantly influenced microbiome structure (Fig. 2A and B, PERMANOVA; Table 1A and B). Further, small (R^2^ ≤ 0.05) effects of disease metrics and meadow characteristics accounted for 20% to 34% of the total observed variation in microbial community composition when summed together for eelgrass (Table 1A) and water (Table 1B), respectively. Randomly shuffling the order of factors in our model did not change model outcomes (Table S1A–C in the supplemental material). Effects detected by PERMANOVA can be due to differences in location or dispersion (variance) in ordination space (46). Thus, we also tested for differences in dispersion of microbial communities. We found that dispersion partially explained differences among geographic regions in eelgrass and seawater microbial community composition (PERMDISP, permutation test of multivariate dispersions, F = 6.121 and 6.863, *P* = 0.001 for each analysis, respectively). Differences in the variance (dispersion) of microbial community composition was not detected with lesion status (PERMDISP, F = 0.046, *P* = 0.829) or tissue type (PERMDISP, F = 0.487, *P* = 0.496). Despite correlations among some explanatory variables (Table S2), our PERMANOVA models for eelgrass and seawater were improved by inclusion of all factors. Dropping factors resulted in more variation observed in the residuals, rather than in the remaining explanatory variables.

**TABLE 1:** Permutation multivariate analysis of variance model output for eelgrass green tissue samples (from both lesioned and nonlesioned leaves) and seawater

Factor	df	Sum of squares	R^2^	*F*	Pr(>F)
Eelgrass green tissue samples A					
Lesion prevalence	1	2.27	0.027	9.43	0.001
Lesion severity	1	1.56	0.019	6.51	0.001
Lesion status	1	0.66	0.008	2.74	0.001
Tidal height	1	0.63	0.008	2.63	0.001
Leaf area	1	1.74	0.021	7.23	0.001
Shoot density	1	1.58	0.019	6.56	0.001
Sheath length	1	0.74	0.009	3.08	0.001
Longest leaf length	1	0.99	0.012	4.11	0.001
SST	1	1.94	0.023	8.08	0.001
Thirty-day maximum SST	1	3.08	0.037	12.82	0.001
Thirty-day minimum SST	1	1.08	0.013	4.47	0.001
Region	5	8.90	0.107	7.40	0.001
Region:Meadow	17	14.38	0.173	3.52	0.001
Residual	181	43.53	0.524	NA	NA
Total	214	83.08	1.000	NA	NA
Seawater B					
Lesion prevalence	1	0.75	0.032	5.04	0.001
Lesion severity	1	1.29	0.054	8.67	0.001
Leaf area	1	1.10	0.046	7.36	0.001
Shoot density	1	0.77	0.032	5.16	0.001
Sheath length	1	0.57	0.024	3.84	0.001
Longest leaf length	1	0.84	0.035	5.60	0.001
SST	1	0.92	0.039	6.18	0.001
Thirty-day maximum SST	1	0.91	0.038	6.11	0.001
Thirty-day minimum SST	1	1.03	0.043	6.92	0.001
Region	5	3.74	0.157	5.01	0.001
Region:Meadow	9	4.87	0.204	3.62	0.001
Residual	47	7.02	0.295	NA	NA
Total	70	23.83	1.000	NA	NA

10.1128/msystems.00224-22.1 TABLE S1 Permutation multivariate analysis of variance model output for Bray Curtis dissimilarity differences between microbial communities of green tissue samples (from lesioned and nonlesioned leaves). The order of predictor variables is randomly shuffled three times. Download Table S1, XLSX file, 0.01 MB. Copyright © 2022 Beatty et al. 2022 Beatty et al. https://creativecommons.org/licenses/by/4.0/ This content is distributed under the terms of the Creative Commons Attribution 4.0 International license.

10.1128/msystems.00224-22.2 TABLE S2 Significant correlations between predictor variables used in analyses of microbial beta diversity. Download Table S2, XLSX file, 0.01 MB. Copyright © 2022 Beatty et al. 2022 Beatty et al. https://creativecommons.org/licenses/by/4.0/ This content is distributed under the terms of the Creative Commons Attribution 4.0 International license.

**FIG 3:**
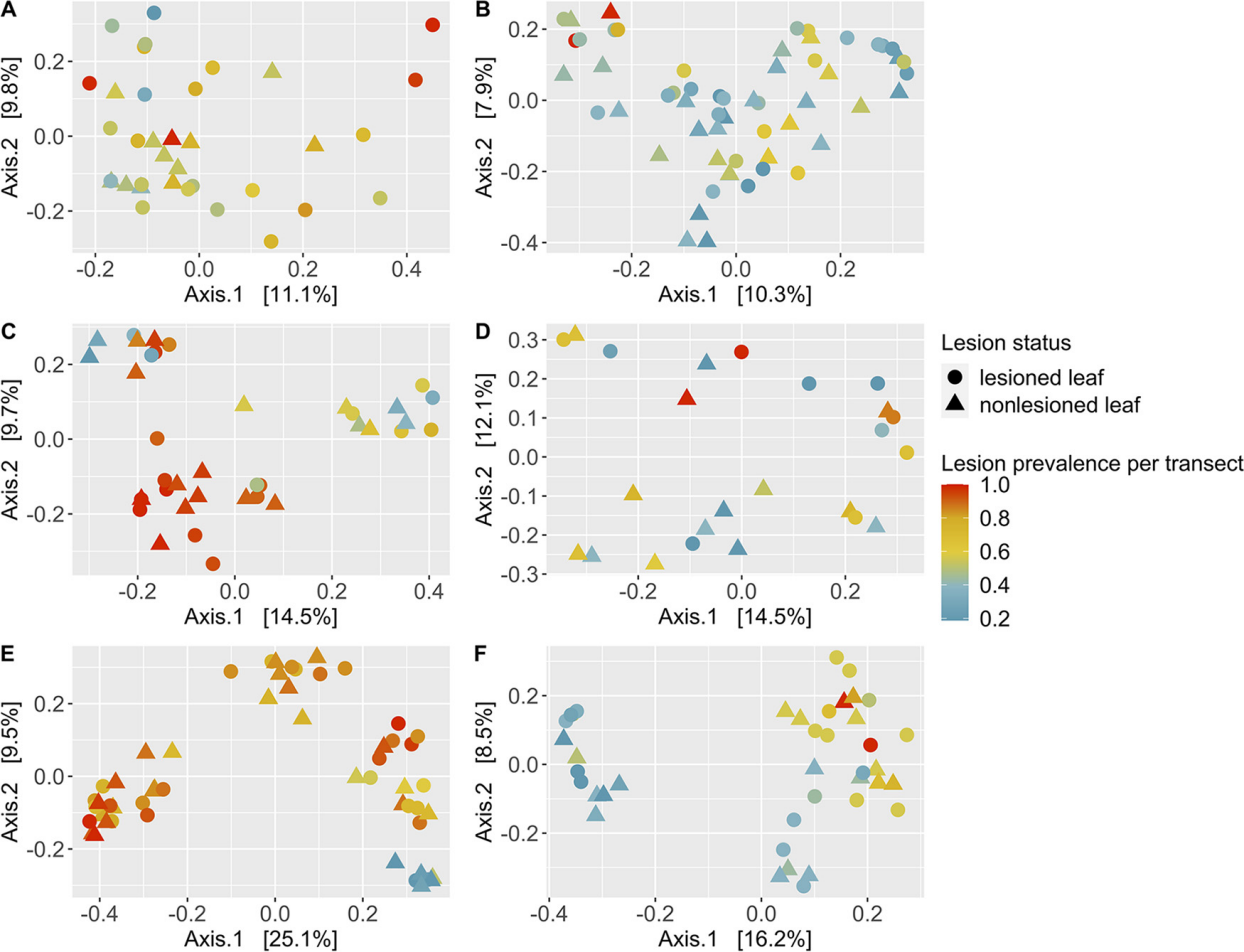
Effects of wasting disease lesion prevalence within meadows on microbial community composition across regions (A, Alaska; B, British Columbia; C, Washington; D, Oregon; E, Bodega; F, San Diego). We performed principal coordinate analysis on matrices of Bray Curtis dissimilarity in microbial community composition from green tissue samples (including lesioned and nonlesioned eelgrass leaves) for each region separately. Wasting disease lesion prevalence explained between 3 to 11 percent of microbial community composition across regions (minimum to maximum effect sizes correspond to British Columbia, B, and Bodega, E, regions, respectively). Lesion status, the presence or absence of lesions, explained two to six percent of microbial community composition (minimum to maximum effect sizes correspond to Washington, C, and Oregon, D, regions, respectively). Wasting disease lesion prevalence and lesion status contributed significantly to microbial community composition (PERMANOVA, *P* < 0.05; Table S3A–F), except for Oregon (lesion prevalence) and Washington (lesion status). Sequence variant tables were rarefied to 4,660 sequences per sample prior to calculating Bray Curtis dissimilarity and principal coordinate analysis.

Due to large regional differences in microbial community composition, we tested the hypothesis that variation explained by wasting disease may be greater among sites within each region rather than among sites across all regions that differ in local conditions. We found support for this hypothesis. Lesion prevalence per transect explained between 3 to 11 percent of microbial community composition within regions (Fig. 3; Table S3A–F; minimum to maximum variation explained correspond to BC, Fig. 3B, and BB, Fig. 3E, respectively) compared to only three percent of community composition across regions (Table 1A). Further, lesion status explained two to six percent of microbial community composition within regions (Fig. 3; Table S3A–F; minimum to maximum variation explained correspond to WA, Fig. 3C, and OR, Fig. 3D, regions, respectively) compared to one percent of community composition across regions (Table 1A). Lesion prevalence per transect and lesion status contributed significantly to microbial community composition within most regions (*P* < 0.05; Table S3A–F), except in OR for lesion prevalence and in WA for lesion status. Greater variability in microbial community composition was observed among green tissue samples from lesioned leaves compared to nonlesioned leaves in AK (PERMDISP, F = 7.4478, *P* = 0.014), but not in any other region (*P* > 0.05).

10.1128/msystems.00224-22.3 TABLE S3 Permutation multivariate analysis of variance model output for Bray Curtis dissimilarity between microbial communities of green tissue samples (from lesioned and nonlesioned leaves) for (A) Alaska, (B) British Columbia, (C) Washington, (D) Oregon, (E) Bodega, and (F) San Diego. Download Table S3, XLSX file, 0.02 MB. Copyright © 2022 Beatty et al. 2022 Beatty et al. https://creativecommons.org/licenses/by/4.0/ This content is distributed under the terms of the Creative Commons Attribution 4.0 International license.

### Alpha diversity results.

**FIG 4:**
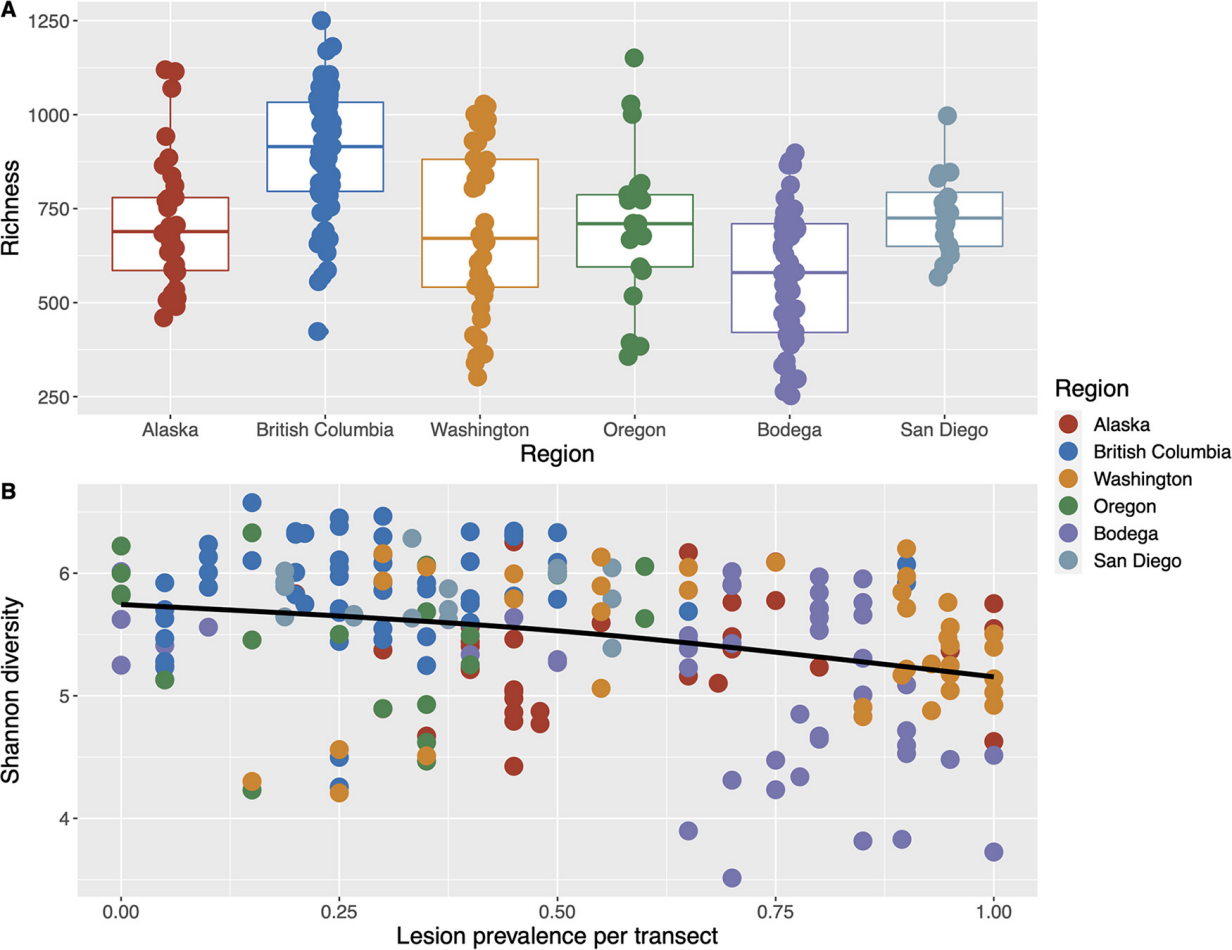
Eelgrass microbial alpha diversity varies by region (A) and declines with wasting disease lesion prevalence (B). Eelgrass sequence variant (A) richness box plots by region (generalized additive mixed effects model, F = 2.83, *P* = 0.017), and (B) Shannon diversity plotted by wasting disease lesion prevalence per transect with a thin plate regression spline smoothed line (F = 3.33, *P* = 0.012). Samples include all green tissue samples (from both lesioned and nonlesioned leaves). Model output can be found in Table S4A–B.

Sequence variant richness among all green tissue samples (including both lesioned and nonlesioned leaves) differed by geographic region (Fig. 4A; Table S4A, generalized additive mixed effects model, F = 2.83, *P* = 0.017), and Shannon diversity was negatively correlated with lesion prevalence per transect across regions (Fig. 4B; Table S4B, F = 3.33 *P* = 0.012). We excluded colinear variables (Table S2, 30-day maximum and minimum SST, leaf area, longest leaf length) from the generalized additive mixed effects model, due to additive model sensitivity to collinearity among factors. Further, in separate models, we found no difference in sequence variant richness or Shannon diversity with tissue type, i.e., between lesion tissue and adjacent green tissue of lesioned leaves (Table S4C and D). We found sequence variant richness and Shannon diversity of seawater microbial communities to be positively correlated with eelgrass sheath length per transect (Fig. S3A and C at https://doi.org/10.6084/m9.figshare.20097311; Table S4E and F, b = 0.72, F = 28.81, *P* < 0.001; b = 0.67, F = 26.13, *P* < 0.001) and negatively correlated with lesion severity per transect (Fig. S3B and D at https://doi.org/10.6084/m9.figshare.20097311; Table S4E and F, linear mixed effects model, b = −0.32, F = 11.13, *P* = 0.005; b = −0.24, F = 4.99, *P* = 0.044).

10.1128/msystems.00224-22.4 TABLE S4 Alpha diversity model output on eelgrass and water samples. Generalized additive mixed effects model output for eelgrass green tissue samples from lesioned and nonlesioned leaves sequence variant richness (A) and Shannon diversity (B), with smoothing function for wasting disease lesion prevalence and random effect of meadow location. Generalized additive mixed effects model output on lesion and adjacent green tissue sample sequence variant richness (C) and Shannon diversity (D), with a random effect of meadow location and smoothing function for sheath length for the richness model and smoothing function for sheath length and wasting disease lesion prevalence for the Shannon diversity model. Linear mixed effects model output for seawater sequence variant richness (E) and Shannon diversity (F), with exponential variance structure for lesion severity and sea surface temperature and a random effect of meadow location. Download Table S4, XLSX file, 0.01 MB. Copyright © 2022 Beatty et al. 2022 Beatty et al. https://creativecommons.org/licenses/by/4.0/ This content is distributed under the terms of the Creative Commons Attribution 4.0 International license.

### Random forest results.

**FIG 5:**
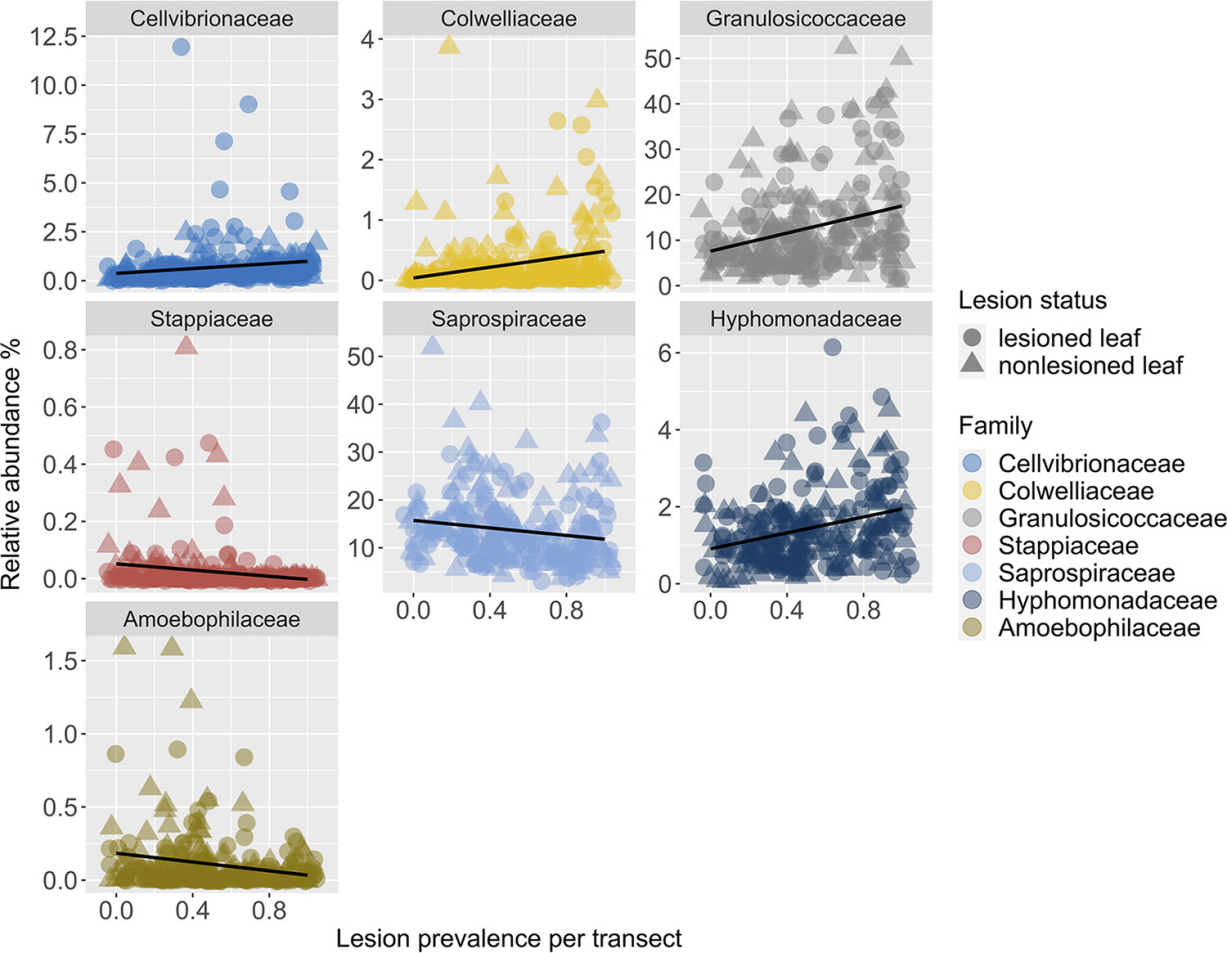
Top seven bacterial families from green tissue samples (on both lesioned and nonlesioned leaves) that are predictive of wasting disease lesion prevalence (random forest regression, model correlation: *R* = 0.75, model fit: R^2^ = 0.56, *P* = 4.22E-08). Family groups are plotted in order of their importance scores in the model from top left most important, Cellvibrionaceae, to bottom right least important, Amoebophilaceae. We rarefied sequence tables to 4,660 sequences per sample prior to collapsing taxonomy to the family level and calculating relative abundances. Taxonomic classification, correlation, and *P* values for each family can be found in Table S5A.

We identified bacterial families (Fig. 5; Table S5A, random forest regression, *R* = 0.75, R^2^ = 0.56, *P* = 4.22E-08) on green tissue samples from lesioned and nonlesioned leaves that are predictive of lesion prevalence per transect across regions. We tested for correlations between lesion prevalence and relative abundances of the top 10 predictive bacterial families (Fig. 5) and found seven out of 10 significant correlations between predictive taxa and lesion prevalence (Table S5A). Given that lesion status could obscure our ability to detect significant correlations between bacterial taxa and lesion prevalence, we tested for correlations of bacterial taxa on green tissue samples from lesioned and nonlesioned leaves separately. By doing so, we identified two additional significant correlations, a negative relationship between Methylophilaceae bacteria relative abundance from green tissue on nonlesioned leaves and lesion prevalence per transect (Table S5B) and a negative relationship between Rickettsiaceae bacteria relative abundance from green tissue on lesioned leaves and lesion prevalence per transect (Table S5C).

10.1128/msystems.00224-22.5 TABLE S5 Top 10 families (A) from green tissue samples of lesioned and nonlesioned eelgrass leaves that are predictive of wasting disease lesion prevalence from random forest regression (R = 0.75, R^2^ = 0.56, *P* = 4.22E-08). Importance values indicate the rank of importance of families from random forest regression. We also tested for correlations between families from nonlesioned leaves (B) and lesioned leaves (C) separately with lesion prevalence after failing to detect a significant correlation for 3 of 10 families when we tested both nonlesioned and lesioned leaves together (A). Download Table S5, XLSX file, 0.01 MB. Copyright © 2022 Beatty et al. 2022 Beatty et al. https://creativecommons.org/licenses/by/4.0/ This content is distributed under the terms of the Creative Commons Attribution 4.0 International license.

We identified bacterial families (Fig. S4 at https://doi.org/10.6084/m9.figshare.20097335.v1; Table S6A, random forest regression, *R* = 0.92, R^2^ = 0.85, *P* = 1.94E-18) on green tissue samples from lesioned and nonlesioned leaves that are predictive of 30-day maximum SST across regions. We tested for correlations between 30-day maximum SST and relative abundances of the top 10 predictive bacterial families and found 8 out of 10 significant correlations between predictive taxa and 30-day maximum SST, respectively (Table S6A). Given that lesion status could obscure our ability to detect significant correlations between bacterial taxa and 30-day maximum SST, we tested for correlations of bacterial taxa on green tissue samples from lesioned and nonlesioned leaves separately. However, we did not identify any additional significant correlations by analyzing lesioned and nonlesioned leaves separately (Table S6A–C). Two of eight families identified as significant predictors by random forest analysis of 30-day max SST, Saprospiraceae and Amoebophilaceae, are also identified by random forest as significant predictors of wasting disease lesion prevalence. However, only the Amoebophilaceae family exhibited negative relationships with both temperature and lesion prevalence.

10.1128/msystems.00224-22.6 TABLE S6 Top 10 bacterial families (A) from green tissue samples of lesioned and nonlesioned eelgrass leaves that are predictive of 30-day maximum SST from random forest regression (R = 0.92, R^2^ = 0.85, *P* = 1.94E-18). Importance values indicate the rank of importance of families from random forest regression. We also tested for correlations between families from nonlesioned leaves (B) and lesioned leaves (C) separately with 30-day maximum SST after failing to detect a significant correlation for 2 of 10 families when we tested both nonlesioned and lesioned leaves together (A). Download Table S6, XLSX file, 0.01 MB. Copyright © 2022 Beatty et al. 2022 Beatty et al. https://creativecommons.org/licenses/by/4.0/ This content is distributed under the terms of the Creative Commons Attribution 4.0 International license.

### Differential relative abundance results.

**FIG 6:**
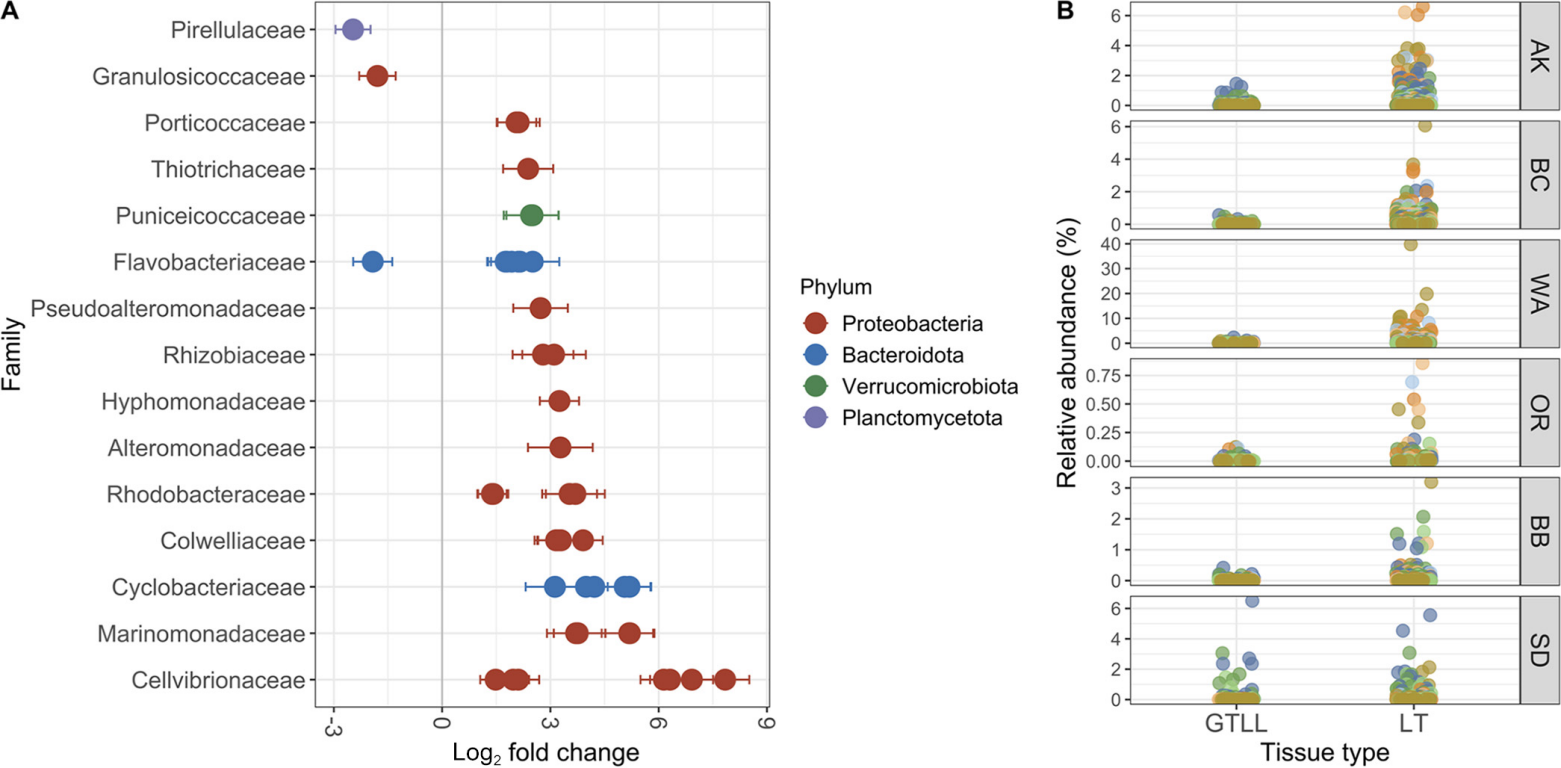
Bacterial sequence variants that are enriched or depleted in lesion tissue (LT) relative to adjacent green tissue from lesioned leaves (GTLL). (A) Log_2_ fold change (±SE) in relative abundances of sequence variants that are present at significantly higher or lower relative abundances in lesion tissue compared to adjacent green tissue. Sequence variants are plotted by family, and color corresponds to phylum. (B) Relative abundances of the seven unidentified Cellvibrionaceae sequence variants enriched in lesion tissue (LT) compared to adjacent green tissue from lesioned leaves (GTLL) plotted across regions. Relative abundances were calculated from tables after rarefying to 4,660 sequences per sample for consistency of taxa relative abundances across figures. Color corresponds to unique sequence variants; full taxonomic identification and fold differences can be found in Table S7A.

We tested for sequence variants and families that differ in relative abundances within lesioned leaves between lesion tissue and adjacent green tissue (i.e., “tissue type”) and between green tissue samples from lesioned and nonlesioned leaves (i.e., “lesion status”), using DESeq2. We found 43 unique sequence variants that differed in relative abundance with tissue type between lesion tissue and green tissue adjacent to lesions (–2.5 to +7.8 log_2_ fold change, Fig. 6; Fig. S5 at https://doi.org/10.6084/m9.figshare.20097341.v1; Table S7A). A sequence variant belonging to the Cellvibrionaceae family exhibited the greatest (7.8 log_2_ fold) increase in relative abundance from green tissue at the leading edge of infection to brown lesioned tissue. We tested for differences in relative abundances of bacterial families between lesion and adjacent tissue and identified 21 bacterial families that differed between lesion and adjacent tissue (–1.3 to 3.5 log_2_ fold change, Fig. S6 at https://doi.org/10.6084/m9.figshare.20097344.v1; Table S7B). Seventeen of the 21 bacterial families that differ between lesion and adjacent tissue are present in rarefied tables (Fig. S6 at https://doi.org/10.6084/m9.figshare.20097344.v1). We tested for differences in bacterial families on green tissue between leaves with and without lesions (i.e., with “lesion status”) and found six bacterial families that differed by −1.2 to +1.2 log_2_ fold in relative abundance (Fig. S7 at https://doi.org/10.6084/m9.figshare.20097347.v1; Table S8). Four of the six bacterial families that differed with lesion status occurred in rarefied tables (Fig. S7 at https://doi.org/10.6084/m9.figshare.20097347.v1). However, no sequence variants differed significantly with lesion status.

10.1128/msystems.00224-22.7 TABLE S7 Sequence variants (A) and families (B) from lesioned leaves that differ in relative abundances between lesion and adjacent green tissue (i.e., with tissue type) from Deseq2 analysis. Download Table S7, XLSX file, 0.01 MB. Copyright © 2022 Beatty et al. 2022 Beatty et al. https://creativecommons.org/licenses/by/4.0/ This content is distributed under the terms of the Creative Commons Attribution 4.0 International license.

10.1128/msystems.00224-22.8 TABLE S8 Bacterial families that differ in relative abundances between green tissue from lesioned and nonlesioned leaves (i.e., with lesion status) from Deseq2 analysis. Download Table S8, XLSX file, 0.01 MB. Copyright © 2022 Beatty et al. 2022 Beatty et al. https://creativecommons.org/licenses/by/4.0/ This content is distributed under the terms of the Creative Commons Attribution 4.0 International license.

## DISCUSSION

By sampling across 23° latitude, we aimed to understand how eelgrass microbial diversity varies with wasting disease. We find (i) large geographic differences in both seawater and phyllosphere microbial communities, but despite this variation, wasting disease metrics are linked to microbial community composition; (ii) that Cellvibrionaceae bacteria, known plant cellulose degraders, are found at low relative abundances on asymptomatic tissue and are enriched in lesion tissue across regions in our study area; (iii) that microbial diversity on asymptomatic phyllosphere tissue declines with rising lesion prevalence within meadows, whereas seawater microbial diversity declines with lesion severity within meadows; and (iv) that predictable changes in the relative abundances of specific taxa occur with rising lesion prevalence within meadows and these taxa differ from those that are correlated with warming water temperatures. These findings suggest that consideration of phyllosphere microbiomes will increase our understanding of the variable outcomes of infection and possibly improve models that forecast disease outbreaks.

### Effects on microbial community composition.

Microbial community composition differs strongly by geographic region for both eelgrass and seawater. These results are consistent with earlier findings that eelgrass leaf and surrounding water microbial communities exhibit similar spatial turnover in microbial community composition across large spatial scales (34). Turnover of microbial communities over a large geographic range may reflect changes in physical, chemical, or biotic drivers (55–57). Further, physiological and morphological differences within and across eelgrass populations (58, 59) may interact with extrinsic variables like temperature and nutrients to drive microbiome structure (60). Indeed, temperature and morphological features of our eelgrass meadows like shoot length, longest leaf length, and shoot density are linked to eelgrass community composition in our global model. Similar links between eelgrass morphological features and seawater microbial communities in our study may reflect some unmeasured variable, like nutrients or light, that can affect plant as well as microbial productivity in these shallow nearshore environments. Despite the large effects of geography in our study, we detect differences in microbial community composition with disease, including enrichment of bacterial families and sequence variants in lesion tissue across regions.

### Enrichment of specific bacterial taxa within lesion tissue.

We identified bacterial families and sequence variants that are consistently associated with lesions across regions in our study, despite differences in local conditions. Thus, these bacteria could affect the fate of infected plant tissue by facilitating or inhibiting development of lesions. Forty-three sequence variants differed in relative abundance between lesion tissue and green tissue at the leading edge of infection. Higher *L. zosterae* density often occurs in green tissue at the leading edge of the infection (17, 61). Thus, we hypothesized that bacteria enriched on green tissue adjacent to lesions may be important for early stages of disease, and those enriched in lesions may influence (later) wasting stages of disease, such as tissue necrosis.

Three sequence variants from families Granulosicocaceae, Flavobacteriaceae, and Pirellulaceae are enriched in green tissue adjacent to lesions relative to lesion tissue. The sequence variant within Granulosicocaceae was identified as a member of the genus *Granulosicoccus*. This genus is common to the *Z. marina* microbiome (31, 62–64) and is found in 16S gene surveys on surfaces of kelp (65) and several species of macroalgae with proposed roles in sulfur cycling (66). Other sequence variants enriched in green tissues adjacent to lesions are classified as *Polaribacter* sp. within the Flavobacteriaceae family and *Blastopirellula* sp. within the Pirellulaceae family. Neither genus has known functional roles in seagrass disease.

In contrast, 40 sequence variants occurred at higher relative abundances in lesion tissue compared to adjacent green tissue. This included seven sequence variants of Cellvibrionaceae and four sequence variants of Colwelliaceae. Both groups, Colwelliaceae and Cellvibrionaceae, were enriched at the family level by ~2- and 3-fold, respectively, in lesion tissue compared to adjacent green tissue (i.e., with “tissue type”). Cellvibrionaceae was also enriched on green tissue adjacent to lesions compared to green tissue from nonlesioned leaves. Taken together, this indicates that sequence variants in the Cellvibrionaceae family are present on asymptomatic tissue from lesioned and nonlesioned leaves but become more dominant community members within lesions. While functional roles of Colwelliaceae and Cellvibrionaceae in wasting disease are not known, Colwelliaceae is associated with lobster shell disease (67) and Cellvibrionaceae are known for degrading cellulose, a component of plant cell walls (68, 69).

Cellvibrionaceae are known for degrading complex plant polysaccharides via cell membrane-bound or secreted extracellular enzymes (68, 69). Thus, enrichment of low relative abundance Cellvibrionaceae from green tissue on nonlesioned leaves to green tissue adjacent to lesions suggests that this group may be involved in early stages of infection and disease by the opportunistic pathogen *L. zosterae*. For example, Cellvibrionaceae may break down plant cell walls or overwhelm plant immune responses to microbial attack (4). Further enrichment of Cellvibrionaceae within lesions compared to adjacent green tissue, combined with their cellulose degrading ability, also suggests a role for this taxon in disease progression. Alternatively, Cellvibrionaceae could be opportunistic saprophytes (4, 69), increasing in response to virulence of *L. zosterae* and available dead plant tissue. However, the enrichment of plant tissue degraders that spatially precede lesion development points to their potential facilitation of wasting disease infection and progression. Follow-up studies are needed to explore temporal changes in the phyllosphere microbiome, but high relative abundances of Cellvibrionaceae within lesion tissue, up to 61% of community composition in some cases, suggest an important functional role in wasting disease progression. More generally, our findings indicate that a holistic approach to investigating the pathogenic microbiome of wasting disease (3, 4) could improve our understanding of variable rates of infection, disease progression, and plant survival (22, 24, 70).

### Declines in microbial diversity on asymptomatic tissue with rising disease prevalence within meadows.

We observed declines in microbial Shannon diversity on asymptomatic green tissue samples from lesioned and nonlesioned leaves that coincided with increases in the prevalence of wasting disease lesions within meadows across 23° latitude. In coral, declines in microbial diversity temporally precede development of white syndromes (71). Reduced host-associated microbiome diversity may render hosts more susceptible to microbial invasion or proliferation of low abundance opportunistic pathogens (72, 73). Indeed, high soil and rhizosphere microbial diversity promotes suppression of some plant pathogens in terrestrial systems due to resource and interference competition (72, 74). Thus, declining phyllosphere microbial diversity may be an early indicator of reduced biotic resistance to opportunistic pathogen *L. zosterae* in eelgrass beds. Alternatively, declining microbial diversity could be a consequence of increasingly dominant pathogenic or saprophytic taxa on asymptomatic tissue, or alteration of resource availability. We similarly observed modest declines in seawater microbial diversity that coincided with increasing wasting disease lesion severity within meadows. How these two variables are linked is not clear at this time; it is possible a third variable not measured in our study, like light or nutrients, could be driving changes in seawater microbial diversity and lesion severity within meadows.

Declines in microbial alpha diversity associated with host stress and disease can be followed by blooms of specific or nonspecific microbial groups (75–77). However, we did not detect greater microbiome variability (dispersion) among eelgrass samples with lesion status or tissue type. This suggests deterministic rather than stochastic or nonspecific blooms of bacteria following infection and development of lesions. Taken together, our findings show declines in microbial diversity are coupled with rising wasting disease prevalence, but this process likely occurs with predictable changes in eelgrass microbiome composition.

### Microbial predictors from asymptomatic tissue of disease prevalence within meadows.

Bacteria may act as early warning indicators of plant stress or disease due to their capacity to respond quickly to changing environmental and biotic conditions (78, 79). We tested whether bacterial families among green tissue samples from lesioned and nonlesioned leaves could predict wasting disease lesion prevalence within meadows across regions. We identified multiple taxa whose occurrence and relative abundances combined can predict lesion prevalence (random forest regression model, *R* = 0.75). Colwelliaceae increased in relative abundance predictably with increasing lesion prevalence within meadows. Colwelliaceae are associated with lobster shell disease (67), with eelgrass experimentally exposed to nutrients (63), and water samples amended with dissolved organic matter (80). Further, Colwelliaceae are enriched in lesion tissue compared to adjacent green tissue in our study. Thus, Colwelliaceae may play a role in the wasting phase of disease, possibly by responding to release of dissolved organic matter (80) during the breakdown of plant tissues, and also act as early warning indicators in green tissue prior to appearance of symptomatic lesions. Interestingly, increases in the relative abundances of Granulosicocceae, Cellvibrionaceae, and Hyphomonadaceae among green tissue samples are also predictive of increasing lesion prevalence within meadows. Members of Granulosicocceae and Hyphomonadaceae are epiphytes of eelgrass and kelp (62, 81, 82); functional roles of these taxa in eelgrass microbiomes are not known. Cellvibrionaceae bacteria, as discussed earlier, are common degraders of plant cellulose and complex polysaccharides (68, 69). Predictable increases of Cellvibrionaceae on asymptomatic tissue samples with increasing prevalence of wasting lesions within meadows further supports their potential role in promoting or opportunistically exploiting *L. zosterae* infection during early stages of disease.

### Temperature effects on disease prevalence and microbiome structure.

Warming is hypothesized to disrupt beneficial microbiomes and their functions within marine habitat forming species, like eelgrass, and may contribute to disease outbreaks (2, 6, 83). Indeed, warm thermal anomalies are linked to higher prevalence of wasting disease along our latitudinal gradient (27) and at our San Juan Island, WA sites (23). This may result from cumulative or interactive effects of warming on plant or pathogen physiology (59, 83, 84). Warming can limit eelgrass growth (59) and promote *L. zosterae* growth (28). Further, eelgrass populations can be locally adapted and show plasticity in response to thermal stress (85, 86). However, warming-induced disruptions to eelgrass microbiomes and potential effects on disease dynamics have yet to be explored. We found that maximum sea surface temperature (SST) over a 30-day window prior to sampling and SST on the day of sampling explained four and two percent of phyllosphere microbiome composition, respectively. Variation explained by 30-day maximum SST suggests that temperature effects on phyllosphere microbiomes may be modulated partially through changes in host physiology, stress, or immune responses (84). We similarly observed effects of SST on the day of sampling and 30-day maximum and minimum SSTs on seawater microbiomes. Thus, warm temperatures during the summer growing season (30 days prior to sampling) alter phyllosphere microbiomes, in addition to enhancing the prevalence of wasting disease lesions (27). However, the microbial taxa that are correlated with warmer 30-day maximum temperatures differ from those that are correlated with wasting disease prevalence, except for one family, Amoebophilaceae, that is negatively correlated with both warmer temperatures and higher disease prevalence. Amoebophilaceae is associated with coral disease (87) but has no known functions in eelgrass microbiomes. Thus, correlations between phyllosphere microbial taxa and disease do not appear to be confounded by temperature effects on the microbiome. Direct interactions between eelgrass, *L. zosterae*, and bacteria likely drive most of the microbial differences we observed between tissue samples, though it is still unclear the extent to which disruptions of eelgrass microbiomes precede, follow, or co-occur with blooms in *L. zosterae*. Different microbial sequence variants likely fit in each of these categories, as described in some detail for Cellvibrionaceae, above. Temporal investigations of host–pathogen–microbiome interactions under thermal stress may help uncover the order and direction of these potentially complex interactions, which will be critical to understanding disease dynamics under climate warming scenarios.

### Conclusion.

We detected declines in alpha diversity and predictable increases in the relative abundances of Cellvibrionaceae, Colwelliaceae, and Granulosicoccaceae on asymptomatic eelgrass phyllosphere microbiomes with rising wasting disease prevalence across coastlines (spanning 3,100 kilometers) in the Pacific Northeast. While functions of Colwelliaceae and Granulosicoccaceae are not known, Cellvibrionaceae are known for degrading plant cellulose. Further, Cellvibrionaceae are enriched in lesioned versus adjacent green tissue and nonlesioned leaves. Cellvibrionaceae and other opportunistic bacteria may aid *L. zosterae* in overwhelming plant immune responses or contribute to lethality of wasting disease on plants by increasing the rate or extent of degradation of host tissues. These findings suggest that some commensal bacteria found at low relative abundances on asymptomatic tissue like Cellvibrionaceae (~0.5–1% mean relative abundance on green tissue from nonlesioned and lesioned leaves, respectively) facilitate rather than hinder wasting disease. This parallels recent experimental findings that knocking back eelgrass-associated bacteria with antibiotics or dilute bleach prior to inoculation with *L. zosterae* reduces lesion severity (Graham et al., submitted for publication). Pathogens often act in concert with other members of the microbiome (3); inclusion of these members in disease studies will likely improve our ability to predict outcomes of host–pathogen–microbiome interactions across variable landscapes and under future climate change scenarios (83, 88, 89).

## MATERIALS AND METHODS

### Site selection.

We sampled 32 eelgrass meadows across latitudes from 55 to 32° N in the Northeastern Pacific during July and August 2019. This range included six regions (AK=Alaska, BC=British Columbia, WA=Washington, OR=Oregon, BB=Bodega Bay Northern California, SD=San Diego Southern California), with 5–6 meadows per region. The location of each region is AK: N 55° 32' 27.124” W 133° 11' 1.0546", BC: N 51° 48' 30.1469” W 128° 13' 27.2182", WA: N 48° 36' 4.9725” W 122° 59' 56.4203", OR: N 44° 6′ 43.717” W 124° 8′ 22.7337", BB: N 38° 14' 30.3218” W 122° 58' 32.5723", SD: N 32° 47' 37.5929” W 117° 12' 57.1071”. We selected eelgrass meadows within each region that had consistently high cover of eelgrass in recent years; in some cases, meadow locations are part of long-term monitoring programs.

### Transect and meadow-level characteristics.

We sampled six transects in the intertidal area of each meadow. This included three in the upper intertidal (closer to shore) and three in the lower intertidal (further from shore, Fig. 1). We sampled a single eelgrass leaf at 1 m intervals to characterize the prevalence and severity of wasting disease lesions (*n* = 20 leaves per transect; 6 transects per site and 32 total sites; *n* = 3,840 total leaves across regions). We standardized this collection by using the third-youngest leaf from the shoot closest to each sampling point. We characterized the longest leaf and sheath lengths (*n* = 5 individuals per transect), at 4 m intervals. We also quantified shoot density by placing quadrats at 4 m intervals from meter 4 to 16 on the transect. Further sampling details can be found in Aoki et al. (27), including the use of an image classification machine learning tool, Eelgrass Lesion Image Segmentation Application (EeLISA [90]), to quantify the presence or absence of lesions and severity of lesions on leaves. Briefly, this entailed collection of the third-youngest leaf, careful removal of epiphytes, and high-resolution scanning to generate images of each leaf. EeLISA classified the area of each leaf that exhibited lesions versus healthy or senescing tissue. We determined leaf area from EeLISA classification, if the entire leaf was scanned, or by multiplying the length times the width of each leaf if the entire leaf was not scanned. We calculated lesion prevalence per transect as the proportion of leaves with lesions present from the 20 leaves collected along each transect. Thus, values fall on a scale of 0–1. We calculated lesion severity as the ratio of lesioned leaf area divided by total leaf area per leaf and averaged these by transect (20 leaves per transect).

We obtained daily Sea Surface Temperatures (SST) for each eelgrass meadow (1-km resolution) from level four analysis of the Group for High Resolution Sea Surface Temperature (GHRSST) product from NASA’s Jet Propulsion Lab PODAAC portal. We used Multi-Scale Ultra-High Resolution (MUR) products where possible, and if MUR was unavailable, we used Global 1-km SST (G1SST) with multi-scale two dimensional variational (MS-2DVAR) blending algorithm (91). Five meadows (one from Bodega, one from Oregon, and three from San Diego) located within enclosed bays or estuaries precluded our ability to extract pixels from MUR or G1SST. All analyses where SST products are included as predictor variables exclude these five meadows. We obtained SST on the day of sample collection and the maximum and minimum SST from 30 days prior to sample collection with a custom script in R v1.2.5042 (42).

### Microbiome sampling.

We collected *Z. marina* tissue from the 3rd-youngest leaf of individual shoots exhibiting wasting disease lesions and, from nearby (within 1 m), a 3rd-youngest leaf free of lesions from a different shoot at haphazard locations along each transect (Fig. 1). We selected the 3rd-youngest leaf (except for OR, where we sampled the 2nd-youngest leaf) because these leaves allow easy identification of wasting disease lesions, which become more difficult to distinguish from senescing tissue on older leaves. Additionally, 3rd-youngest leaves are still actively growing and so damage to this leaf presumably represents a cost to the plant. Third-youngest leaves occur outside the leaf sheath in OR, unlike other regions, and are covered with epiphytes, precluding our ability to determine lesion status in the field. Because 2nd- and 3rd-youngest leaves in OR did not differ in lesion coverage (27), we decided to include 2nd-youngest leaf samples from OR in our region-wide analyses.

We selected tissue samples from *Z. marina* as described below. *L. zosterae* is an opportunistic endophyte that forms an ectoplasmic net, moving through host tissue by degrading cell walls, with the greatest pathogen density at the leading edge of the infection before tissue browning (17, 61). Thus, we sampled lesion tissue as well as green tissue at the leading edge of lesions to determine how microbiomes may change with lesion development (i.e., effects of “tissue type” on the same leaf). Sampling the leading edge of an infection may allow us to determine early microbial interactors in eelgrass wasting disease versus opportunistic microbes that colonize or increase in abundance following pathogen degradation of host tissue. We also sampled green tissue from a different shoot nearby whose third-youngest leaf did not exhibit lesions (lesion-free leaves) to compare these to green tissue at the leading edge of infection from lesioned leaves (effects of “lesion status”). Lesion status allows us to test how wasting disease, identified by the presence of characteristic lesions on young (nonsenescing) leaves, affects the green phyllosphere microbiome adjacent to lesions. We did not examine all leaves of each shoot for lesions, which would be impractical to identify on older epiphytized and senescing leaves, but only the leaf from which we were sampling. Thus, we do not have samples from individual shoots that are definitively lesion free across all their leaves (disease symptom-free). Identification of lesion-free plants was not possible due to belowground rhizome connections between shoots. However, our sampling of lesion-free young leaves allows for assessment of disease status that likely reflects outcomes for plant fitness as described above. Namely, that younger leaves are still actively growing and so damage to this leaf from wasting disease lesions presumably represents a cost to the plant. If we were unable to find an adjacent (within approximately 1 m) shoot without lesions on the third-youngest leaf or if we were unable to find green tissue adjacent to a lesion due to heavily lesioned leaves, we moved further along the transect and attempted to sample all three tissue types again as described above. To minimize cross-contamination, we wore nitrile or similar gloves and cleaned metal tweezers and scissors with 70% ethanol wipes between each tissue sample. We immediately placed samples into 1.5 mL microcentrifuge tubes containing DNA/RNA shield (Zymo Research Cat. R1100) upon collection.

We also sampled seawater microbial communities to assess whether drivers of eelgrass microbiome structure differ from those of free-living microbial communities in the water column surrounding eelgrass beds. We collected three bottles of water, using 500 mL sterile bottles (VWR Cat. 76299-562) from 3 haphazardly selected locations within the meadow that were approximately 20 m apart. We kept water samples on ice until filtration within 4–6 h of collection with Nalgene analytical filtration units (Cat. 130-4020), which contained 0.22 μm pore size, 47 mm cellulose nitrate filters. Final volumes of water filtered varied (200–500 mL) due to high turbidity at some sites that reduced the rate of filtration. Upon completion of water filtration, the filter was preserved in 2 mL of DNA/RNA shield (Zymo Research Cat. R1100).

### Sample extraction.

Samples were shipped to the University of California, Davis within 3 weeks of sampling and stored at −80°C until processing. We extracted DNA from our samples with ZymoBIOMICS DNA Microprep Kit (Cat. D4301). Upon thawing, we vortexed the 1.5 mL tubes containing leaf tissue in DNA/RNA shield for 60 s and transferred 500 μL of supernatant to a ZymoBIOMICS bead beating tube. We added 500 μL of ZymoBIOMICS lysis solution to the bead beating tube, vortexed tubes for 20 min on a Vortex-Genie 2 with horizontal microtube holder, and performed DNA extractions for phyllosphere samples according to the manufacturer’s instructions following the bead beating step.

To extract DNA from filtered seawater samples, we aseptically cut cellulose nitrate filters into 1–2 mm wide sections, transferred slices plus the 2 mL of DNA/RNA shield used to preserve the filter into two bead beating tubes, and vortexed these on a Vortex-Genie for 20 min. All other DNA extraction steps followed manufacturer instructions, except for final elution of DNA in 40 μL rather than 20 μL of DNase/RNase-free water and further dilution (1 part DNA to 9 parts DNase/RNase-free water) so that DNA concentrations would be similar for both eelgrass tissue and seawater samples, commonly falling between 1 and 10 ng/μL. We processed six negative controls similarly. Four negative controls originated from ZymoBIOMICS DNA/RNA shield and two from sterile cellulose nitrate filters following 100 mL filtration with molecular grade water (Sigma-Aldrich Cat. W4502) preserved in ZymoBIOMICS DNA/RNA shield. Following extraction, negative control samples underwent library prep with all biological samples according to specifications below.

### Library prep and sequencing.

We shipped DNA samples to Dalhousie University's IMR facility for library prep and Illumina MiSeq sequencing according to (92). Briefly, Nextera fusion primers F515 and R926 (93) amplified the V4–V5 region of the 16S rRNA gene with high-fidelity polymerase and 2 μL of template DNA in 25 μL PCR volumes. PCR products from two technical reactions per biological sample were verified with Invitrogen 96-well E-gels. Pooled technical replicates were cleaned and normalized with Invitrogen Sequal-Prep plates. Cleaned and normalized PCR amplicons underwent paired-end 300 bp sequencing on an Illumina MiSeq.

### Data and code availability.

Raw fastq files can be found at the sequencing read archive at NCBI under BioProject number PRJNA802566. Code for this project is archived at https://doi.org/10.5281/zenodo.6228212.
